# Molecular Dynamics Simulation Study of the Selective Inhibition of Coagulation Factor IXa over Factor Xa

**DOI:** 10.3390/molecules28196909

**Published:** 2023-10-02

**Authors:** Hyun Jung Yoon, Sibsankar Kundu, Sangwook Wu

**Affiliations:** 1 Department of Physics, Pukyong National University, Busan 48513, Republic of Korea; hjyoon@pukyong.ac.kr; 2 R&D Center, PharmCADD Co., Ltd., Busan 48792, Republic of Korea; sskundu@gmail.com

**Keywords:** blood coagulation factor, molecular dynamics simulation, selectivity, FIXa, FXa

## Abstract

Thromboembolic disorders, arising from abnormal coagulation, pose a significant risk to human life in the modern world. The FDA has recently approved several anticoagulant drugs targeting factor Xa (FXa) to manage these disorders. However, these drugs have potential side effects, leading to bleeding complications in patients. To mitigate these risks, coagulation factor IXa (FIXa) has emerged as a promising target due to its selective regulation of the intrinsic pathway. Due to the high structural and functional similarities of these coagulation factors and their inhibitor binding modes, designing a selective inhibitor specifically targeting FIXa remains a challenging task. The dynamic behavior of protein–ligand interactions and their impact on selectivity were analyzed using molecular dynamics simulation, considering the availability of potent and selective compounds for both coagulation factors and the co-crystal structures of protein–ligand complexes. Throughout the simulations, we examined ligand movements in the binding site, as well as the contact frequencies and interaction fingerprints, to gain insights into selectivity. Interaction fingerprint (IFP) analysis clearly highlights the crucial role of strong H-bond formation between the ligand and D189 and A190 in the S1 subsite for FIXa selectivity, consistent with our previous study. This dynamic analysis also reveals additional FIXa-specific interactions. Additionally, the absence of polar interactions contributes to the selectivity for FXa, as observed from the dynamic profile of interactions. A contact frequency analysis of the protein–ligand complexes provides further confirmation of the selectivity criteria for FIXa and FXa, as well as criteria for binding and activity. Moreover, a ligand movement analysis reveals key interaction dynamics that highlight the tighter binding of selective ligands to the proteins compared to non-selective and inactive ligands.

## 1. Introduction

Blood coagulation plays a crucial role in maintaining hemostasis. However, deviations from normal coagulation can have severe consequences, leading to a range of coagulation-associated disorders [1,2,3]. Among these disorders, atrial fibrillation stands out as a significant contributor, as it promotes the formation of blood clots, thereby precipitating various clinical pathological conditions, including arterial [4] and venous thrombosis [5], heart attacks, ischemic strokes [6], pulmonary embolisms [7], and cardiac strokes [8]. Collectively, these conditions fall under the category of thromboembolic disorders, which continue to pose substantial threats to both mortality and disability rates in the modern era [1,2,3]. In addressing these disorders, anticoagulants have emerged as the established treatment and can effectively manage thromboembolic disorders and improve patient condition. These medications work by preventing the formation of blood clots or by breaking up existing clots. Anticoagulants can be used to prevent or treat a variety of thromboembolic disorders, including atrial fibrillation, deep vein thrombosis, and pulmonary embolisms.

Recent advancements have led to the approval of several direct oral anticoagulants (DOACs) [9,10] by the FDA which target factor Xa (FXa) and offer a more direct regulation of coagulation pathways. Although DOACs exhibit improved bleeding risk profiles compared to heparin [11] and warfarin [12], which work in an indirect way, they still need to be prescribed with caution. Nevertheless, it is important to note that DOACs such as apixaban [13,14], rivaroxaban [15,16], letaxaban [17], and eribaxaban [18] do not eliminate the risk of bleeding entirely, as they target the common coagulation process pathway. As a result, the search for safer and selective anticoagulants remains a challenging and active area of research. In order to address the partial bleeding risk associated with current anticoagulant therapies, an alternative therapeutic strategy has been proposed to discover agents that can selectively modulate the intrinsic coagulation pathway without interfering with the extrinsic or common pathway [19,20,21,22]. This approach aims to strike a delicate balance between clot formation and blood fluidity. Inhibiting specific coagulation factors within the intrinsic pathway has emerged as an appealing target for thromboembolic disorders, offering the potential to reduce the bleeding risk. One such target is factor IXa (FIXa), situated just upstream of factor Xa (FXa) in the downstream propagation of coagulation [3] (Figure S1 in the Supplementary Materials). This strategy hypothesizes and validates the use of selective FIXa inhibitors that can improve bleeding risk profiles while maintaining a similar efficacy to FXa inhibitors. The underlying principle of this approach lies in selective intrinsic pathway regulation while sparing targets in the extrinsic and common pathways [3].

Selectivity can be achieved through various approaches, including expanding small active molecules to larger compounds with increased interactions, designing analogues with selective interactions, and incorporating the required interactions into lead molecules [23,24,25,26,27]. While Congreve and coworkers [25,28] demonstrated the possibility of growing small molecules with improved selectivity, Eshleman and coworkers [29] emphasized the significance of designing analogues with selective interactions. Additionally, studies by Wacker and coworkers [26,30] highlighted the role of binding site structural differences and amino acid sequence variations to achieve selective inhibition. The pharmacological behavior of a ligand, including its binding, biological activity, potency, and selectivity, is postulated to be influenced by the strength of the protein–ligand interaction [29]. This strength, in turn, depends on the distances between the interaction points between the ligand and protein, which undergo changes during dynamic processes. Thus, the dynamic behavior of these interactions holds significant importance in understanding the pharmacological behavior, such as ligand potency and selectivity [31,32,33]. To investigate the target selectivity of a ligand, we employ molecular dynamics simulation, allowing us to gain valuable insights into the dynamic nature of protein–ligand interactions and its implications on pharmacological properties.

In our previous study [3], we conducted a structure-based investigation utilizing cross docking and pharmacophore-based methods to explore selectivity criteria based on protein–ligand interactions in co-crystal structures. While X-ray co-crystal structures provide valuable insights into the compact and static structures of protein–ligand complexes, their ability to capture the functional dynamics and movements of these complexes is limited. Recognizing that a static structure alone cannot account for functionality, the use of molecular dynamics simulation offers a more realistic approach to studying functional dynamics and movements to understand protein–ligand interactions. In this study, we conducted molecular dynamics simulation to investigate the dynamic behavior of protein–ligand interactions and explore how the interaction strength can help determine selectivity criteria in FIXa and FXa. These findings provide crucial information for understanding the selectivity mechanisms of FIXa and FXa, offering potential avenues for the design of selective inhibitors to target these proteins.

## 2. Results and Discussion

### 2.1. Basic Analysis

A basic analysis was performed on the combined trajectories, which consisted of three sets of 100 ns simulations. Prior to the analysis, the structure of the entire trajectory was aligned to the C_α_ atom of the protein. The complexes’ structural stability during the simulation was evaluated using the RMSD. Across all 22 protein–ligand complexes, the average RMSD values of the protein C_α_ atoms (as shown by the red lines in Figure S2) were approximately 1.5 Å. Notably, differences in the RMSD profiles of the heavy ligand atoms (represented by the black lines in Figure S2) were observed between the ligand datasets. Comparing the FIXa and FXa complexes in (a) and (b) of Figure S2, it can be observed that the RMSD profiles of the active protein–ligand complexes were more stable than those of the complexes with inactive proteins, with lower average RMSD values. This observation is further supported by the comparison of active ligands in Figure S2c and inactive ligands in Figure S2d. The RMSF plot (Figure S3) illustrates the residue flexibility of the heavy chain during the simulation. Similar patterns in the RMSF plots of the protein’s heavy chain were observed in the protein–ligand complexes of FIXa (Figure S3a) and FXa (Figure S3b). The residues in the loop region of the protein exhibited high fluctuations.

### 2.2. Ligand Dynamics

**Figure 1:**
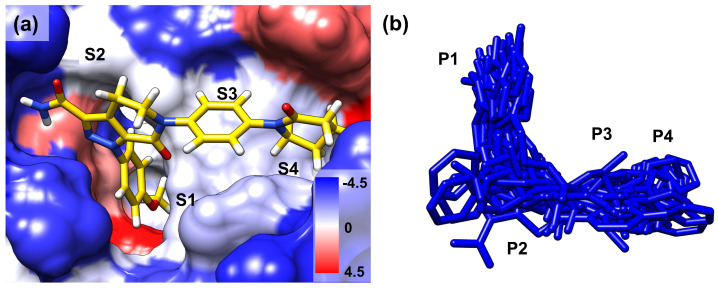
(**a**) The binding mode of the co-crystallized ligand (PDB 2P16) in the binding site of FXa. The surface colors are based on hydrophobicity [34], from blue, which is the most hydrophilic, to white and red, which are the most hydrophobic. S1, S2, S3, and S4 are the binding site subsites. (**b**) All the ligands in the protein–ligand complex are aligned to FIXa. P1, P2, P3, and P4 are the corresponding regions of the ligands that occupy the binding site subsites.

**Figure 2:**
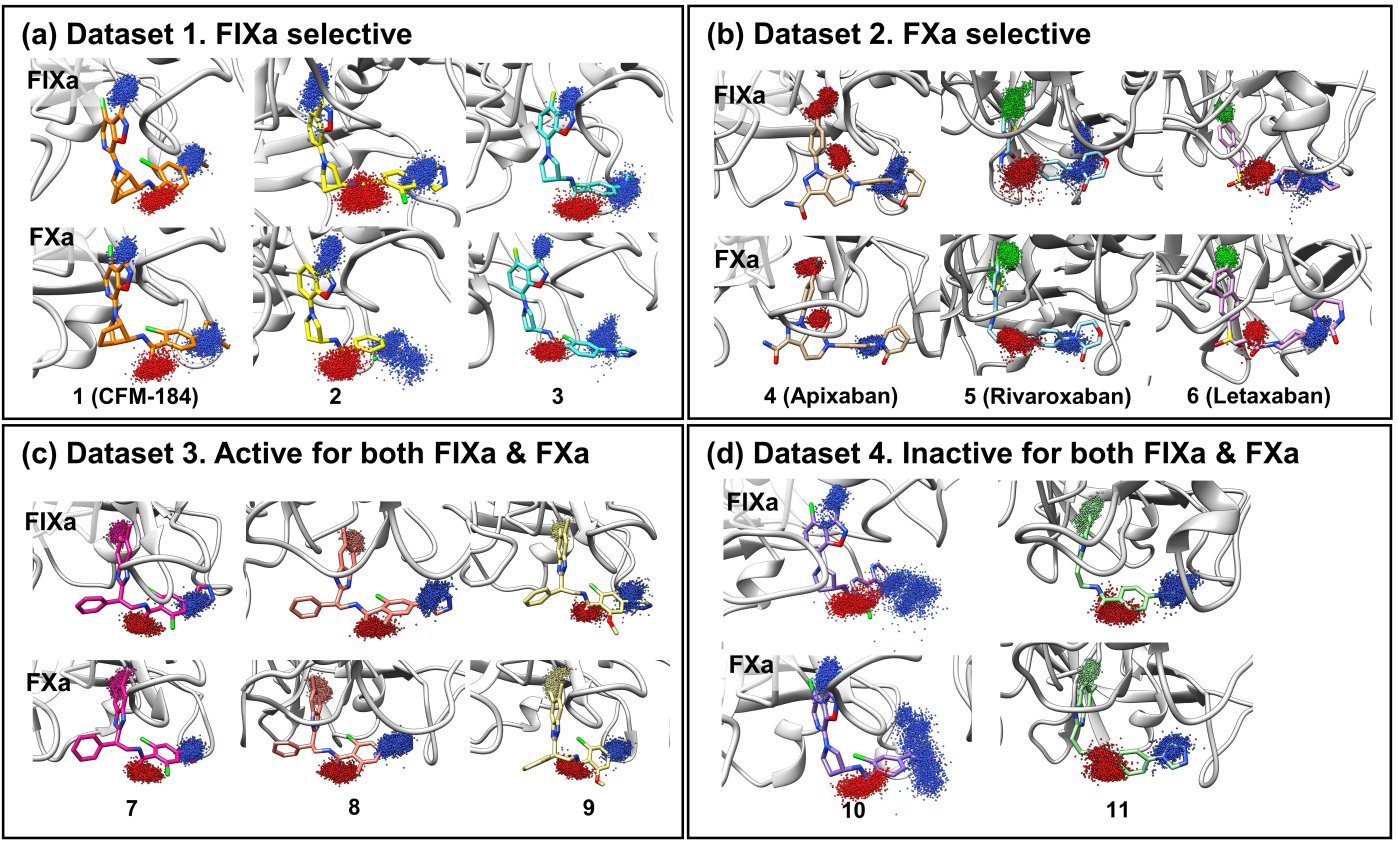
Ligand movement patterns upon binding with FIXa and FXa were evaluated. To assess ligand dynamics, three specific ligand atoms were selected from the P1, P2, and P4 regions of the ligand (blue: nitrogen, red: oxygen, green: chlorine, others: carbon). The atom coordinates were extracted from the entire trajectory of the three replicated 100 ns simulations and are presented in the figure. Each figure shows atomic movement patterns for ligands that are (**a**) FIXa selective, (**b**) FXa selective, (**c**) active for both factors, and (**d**) inactive for both factors.

FIXa and FXa binding sites were divided into several subsites (S1, S2, S3, and S4 (Figure 1b)). The ligands that bind to these sites are also segmented into corresponding fragments (P1, P2, P3, and P4 (Figure 1b)). Ligand dynamics within the binding site were assessed during the MD simulations to obtain information regarding affinity. Atomic coordinates throughout the entire simulation were extracted for three atoms located at P1, P2, and P4. These atom coordinates are presented in Figure 2 to evaluate ligand movement and flexibility. When comparing ligands with selectivity for FIXa or FXa (Datasets 1 and 2), more diffused ligand movements were observed in complexes with non-selective proteins (Figure 2a: FIXa selective and Figure 2b: FXa selective). This difference was particularly pronounced for the selected atoms from P2 and P4, which interact with residues of the S2 and S4 binding subsites (Figure 1a) located on the protein surface. In contrast, atoms from P1, which interact with the S1 site buried inside the protein, exhibited more confined ligand movements. This trend was further supported by a comparison with Dataset 3 (Figure 2c; active for both FIXa/FXa) and Dataset 4 (Figure 2d; inactive for both FIXa/FXa). Ligands in Dataset 3, showing affinity for both FIXa and FXa, displayed stable ligand dynamics characterized by dense atomic coordinates (more confined) throughout the simulation. Conversely, in Dataset 4, P2 and P4 exhibited a higher mobility (more diffused). These findings indicate that the binding of proteins with a lower activity is associated with instability.

### 2.3. Contact Analysis of Protein–Ligand Complex

**Figure 3:**
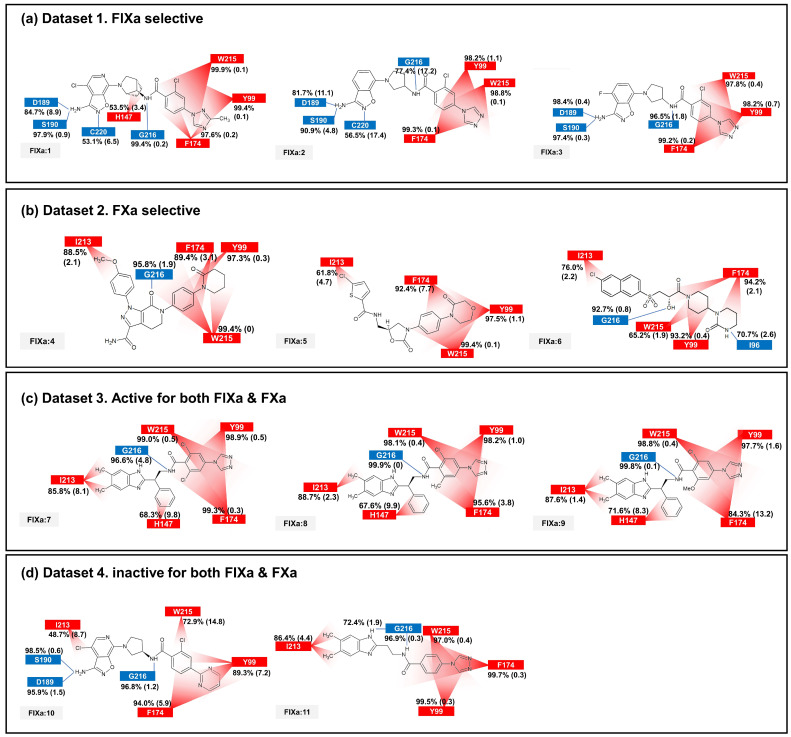
The average and standard deviation (in brackets) of protein–ligand contacts for ligand binding with FIXa. The average contact was calculated by determining interactions with more than 40% contact in each run for protein–ligand complex systems. Hydrogen bonds are marked in blue and hydrophobic contacts are marked in red.

**Figure 4:**
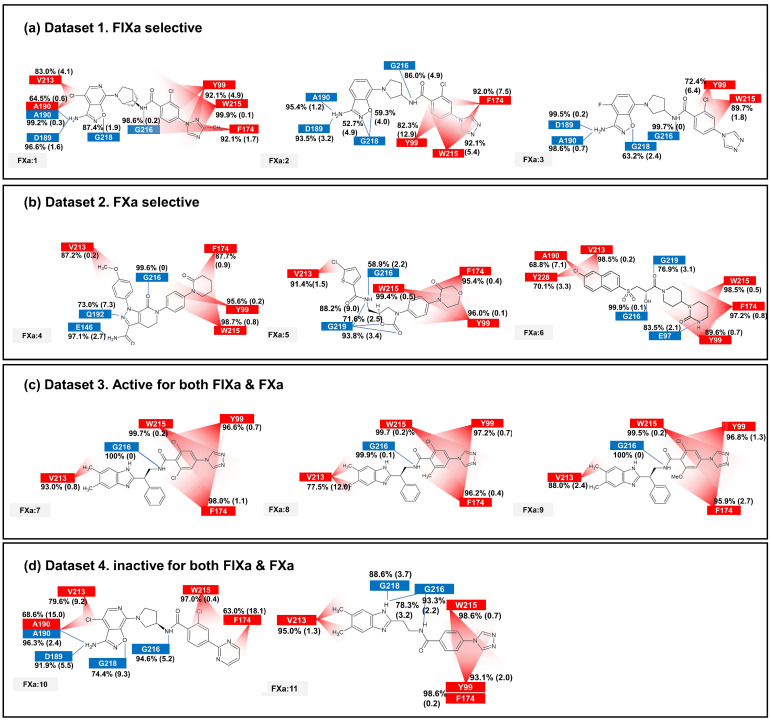
The average and standard deviation (in brackets) of protein–ligand contacts for ligand binding with FXa. The average contact was calculated by determining interactions with more than 40% contact in each run for protein–ligand complex systems. Hydrogen bonds are marked in blue and hydrophobic contacts are marked in red.

The binding affinity to FIXa and FXa was investigated through contact frequency analysis, utilizing the *contactFreq.tcl* script in VMD as illustrated in Figure 3 and Figure 4. The study focused on two types of contacts, namely hydrogen bonds and hydrophobic interactions, throughout the MD simulations. Overall, the majority of the 11 ligands formed hydrogen bonds with G216 and engaged in hydrophobic contacts with Y99, F174, and W215 in both FIXa and FXa complexes. These residues that interact with P4 are recognized as crucial binding sites that form a hydrophobic box [35,36]. Notably, complexes involving an inactive protein exhibited lower hydrophobic contact frequencies with these three residues (Figure 3b,d and Figure 4a,d), thereby influencing the ligand dynamics observed in Figure 2d.

In the FIXa and ligand complexes, hydrophobic contacts were observed between P1 and I213, while hydrogen bonds were formed with D189 and S190 of the S1 subsite (Figure 3). Similarly, in the case of P1 binding to the S1 subsite of FXa, hydrophobic contacts were observed with V213 and hydrogen bonds were formed with D189 and A190 of the S1 subsite (Figure 4). The interaction between the ligand and the S1 subsite is of significant importance when designing inhibitors targeting FIXa and FXa [37,38].

A notable difference was observed when comparing contacts between FIXa and active and inactive ligands (Figure 3a,c and Figure 3b,d, respectively). A hydrophobic contact with H147 at P2 was commonly found in FIXa complexes with **1**, **7**, **8**, and **9** (FIXa:1, FIXa:7, FIXa:8, and FIXa:9 in Figure 3a,c). The interaction between the binding site and P2 of the ligands, which is a flexible region in the FIXa complex, resulted in reduced ligand mobility, thereby stabilizing the bond between P4 and the S4 subsite. Although ligands **2** and **3** (FIXa:2 and FIXa:3 in Figure 3a) exhibited FIXa activity, no hydrophobic contact with H142 was observed. Experimental IC_50_ data (Table 1) confirmed that the activities of ligands **2** and **3** were lower than those of the ligands **7**, **8**, and **9** in Dataset 3 (Figure 3c). These findings are consistent with the previous ligand dynamics analysis (Figure 2).

In the case of the FXa complex, differences were also observed in P2 during contact analysis with active ligands. A hydrogen bond was formed between P2 and a residue in the binding subsite for ligands **4**, **5**, and **6** in Dataset 2 (Figure 4b). Q192 and E146 formed a hydrogen bond in FXa:4, while G219 formed a hydrogen bond with FXa in FXa:5 and FXa:6. These interactions contribute to the stability of the protein–ligand complex. When the ligands from Dataset 1 (**1**, **2**, and **3**) and Dataset 4 (**10** and **11**) were bound to FXa, G218 (identical to G219) formed a hydrogen bond with P1. These ligands exhibit relatively fewer hydrophobic contacts between P4 and the S4 subsite and a lack of activity. These results indicate that the interaction between P2 and S2 is also important in FXa.

### 2.4. Binding Free-Energy Calculation by MM-PBSA

To investigate the binding affinity between ligands and the two proteins, FIXa and FXa, binding free-energy calculations were performed using the last 10 ns of the trajectory from three replicated simulations of each system. After obtaining values from the three repeated simulations, the average value was taken as the result. Figure S4 shows the binding free energy between the ligands and FIXa/FXa. Based on these results, we attempted to evaluate the ligand binding affinity with FIXa and FXa. Although it was not possible to rank the ligands in terms of binding affinity, we were able to determine which protein, FIXa or FXa, exhibited a higher affinity for each ligand. The results were consistent with the experiment, except for the protein–ligand complex bound to **1** and **10**.

Additionally, we assessed the residues with high contribution energies for ligand binding to FIXa and FXa in each system (Tables S1 and S2). Y99, F174, C191, Q192, W215, G216, and C220 exhibited high contribution energies in most of the protein–ligand complexes for both FIXa and FXa. In the contact analysis, we previously identified Y99, F174, W215, and G216 as important residues for binding ligands to FIXa and FXa. Through the analysis of residue contribution energies, it was determined that Q192, C220, and C191, located in the S1 subsite, contribute to the binding of all ligands to FIXa and FXa. In the FXa complex (Table S2), E217 and G218(219) were found to significantly contribute to ligand binding to FXa.

Even though the MM-PBSA method with an implicit solvent model shows some limitations [39,40,41], it is a widely used method to estimate binding free energies [42,43,44]. However, binding free energy estimation is useful to qualitatively evaluate the binding affinity between proteins and ligands. In our study, despite the limited insights into selectivity gained from MM-PBSA protein–ligand complex calculations, key residues were identified by analyzing their residue-wise contributions to ligand–protein interactions.

### 2.5. Binding Site Volume

The binding site volume was calculated to evaluate the binding stability and affinity of the protein–ligand complexes. The calculation was performed at intervals of 1 ns for 100 ns. After obtaining volume values from the three repeated runs, their average value was taken as the result. In this analysis, it was assumed that if the ligand was stably bound to the binding site, a reduction in the volume of the binding site would occur. While variations could arise due to differences in the shape and size of the ligand, it was anticipated that when the same ligand was bound to both FIXa and FXa, we could feasibly differentiate which protein showed a greater binding affinity.

By comparing the binding site volume (as shown in Figure S5), the protein (FIXa or FXa) that binds more stably to the ligand was confirmed. In the complexes involving ligands **1**, **2**, **3**, **8**, **9**, **10**, and **11**, the binding site volume of FXa was observed to be larger than that of FIXa. In the Dataset 2 complexes (comprising ligands **4**, **5**, and **6**), the binding site volume of FIXa was observed to be greater than that of FXa. This observation was consistent with the experimental IC_50_ values, as anticipated. In the complex involving ligand **7** and FIXa/FXa, FIXa exhibited a larger binding site volume than FXa, contradicting the trends observed in other results. This discrepancy resulted from substantial alterations in ligand movement during Run 2 (Frame 2001-3000), as corroborated by the prior RMSD findings (FIXa:7 in Figure S2c). A detailed trajectory analysis confirmed that the root cause was a disrupted interaction between the P2 segment of the ligand and the S2 subsite of the protein. Through this result, it was confirmed that the binding site volume reduced due to tighter and more stable binding in complexes with a protein that has a high binding affinity.

### 2.6. Comparison of Static and Dynamic IFP

In a prior investigation on the selective inhibition of FIXa and FXa, Kundu et al. (2021) primarily focused on molecular modeling studies that exclusively utilized X-ray co-crystal structures. This study predominantly focused on static structural data, without delving into dynamic behavior. In contrast, the current study takes a comprehensive approach, extensively exploring the dynamic aspects of protein–ligand interactions. In this section, we attempted to compare protein–ligand interaction fingerprints (IFPs) from static structural data and dynamic data, called static IFPs and dynamic IFPs, respectively. A dynamic IFP analysis was performed over three replicated 100 ns simulations for each system. The ligands are listed for comparison for dynamic IFPs and static IFPs [3] in Tables S3 and S4 of the Supplementary Materials. To facilitate an effective dynamic IFP analysis, interactions with more than 40% occupancy of hydrogen bonds and pi–pi stacking were considered.

In the dynamic IFPs of FIXa complexes, hydrogen bonds were formed between D189 and S190 with ligands **1** and **2**. Additionally, all ligands exhibited hydrogen bond formation with G216 and displayed pi–pi stacking interactions with Y99, F174, and W215 of FIXa. However, in the static IFPs of FIXa complexes, pi–pi stacking interactions were only observed with F174 and W215, and Y99 was not involved in these interactions. Notably, even ligand **2** did not exhibit pi–pi stacking interactions with any residue of FIXa in the static IFP. Furthermore, pi–pi stacking between H147 and the ligand’s P2 was not detected in the static IFP but was observed for **7** in the dynamic IFP. The dynamic IFPs of the FXa complexes exhibited similar hydrogen bonding interactions in both the dynamic and static IFPs. However, no hydrogen bond was detected between ligand **6** and Q192 of FXa in the dynamic IFP. FXa complex pi–pi stacking interactions differed between the dynamic and static IFPs. In the dynamic IFP, pi–pi stacking interactions occurred in **4** and **5**, although the occupancy was not notably high. No such interactions occurred in ligand **6**. In contrast, the static IFP analysis showed that all ligands had pi–pi stacking interactions, but only with Y99 of FXa.

In the dynamic IFP, dynamically generated pi–pi stacking interactions were identified that were not present in the static IFP. The dynamic IFP results for all datasets regarding interactions other than hydrogen bonds and pi–pi stacking are given in Tables S5 and S6 in the Supplementary Materials.

## 3. Materials and Methods

### 3.1. Preparation of Protein–Ligand Complexes

**Figure 5:**
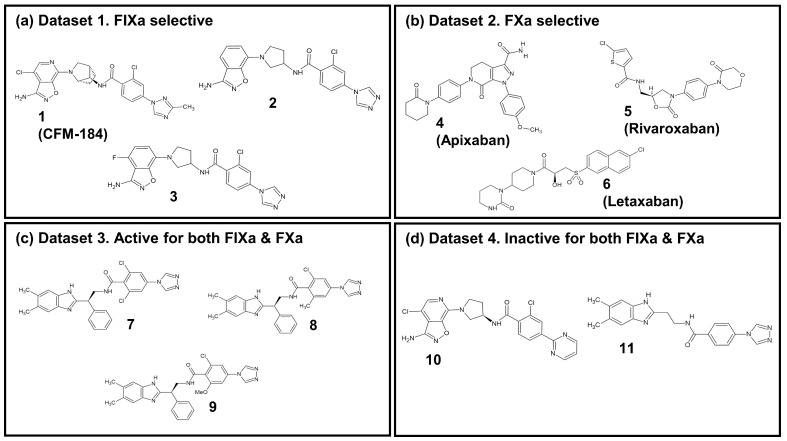
The 2D chemical structure of the 11 ligands.

In this study, an investigation into the selectivity between two coagulation factors, FIXa and FXa, which exhibit structural and functional similarities (Figure S6a) as well as inhibitor binding mode similarities (Figure S6b–d), was performed with eleven ligands. The details of these ligands are listed in Table 1. The ligands were categorized into four groups, as illustrated in Figure 5: (i) Dataset 1: FIXa-selective ligands; (ii) Dataset 2: FXa-selective ligands; (iii) Dataset 3: active ligands for both FIXa and FXa; and (iv) Dataset 4: inactive ligands for FIXa and FXa. Among these, seven co-crystal structures of protein–ligand complexes were obtained from the Protein Data Bank (PDB) (Berman et al., 2000 [45]): compounds **1** (PDB 5TNT [19]), **2** (PDB 5TNO [19]), **7** (PDB 4ZAE [22]), and **11** (PDB 4YZU [20]) bound to FIXa and compounds **4** (PDB 2P16 [13]), **5** (PDB 2W26 [15]), and **6** (PDB 3KL6 [17]) bound to FXa. For compounds **3**, **8**, **9**, and **10**, where co-crystal structures were not available, protein–ligand complex structures were generated by aligning the ligands with the reference co-crystal structures using BIOVIA Discovery Studio [46]. The structures of compounds **3** and **10** were taken from compound **3b** and **7** detailed by Sakurada et al. (2017) [19], respectively, and their ligand structures were generated based on the co-crystal structure of **2**. Similarly, the structures of compounds **8** and **9** were taken from compounds **15** and **17** determined by Zhang et al. (2015) [22], respectively, and their ligand structures were generated using the co-crystal structure of **7**. The resulting aligned ligands were combined with FIXa to form the protein–ligand complex structure. Furthermore, FIXa complexes were aligned with FXa (PDB 2P16), and FXa complexes were aligned with FIXa (PDB 5TNT) to generate the complex structures of each protein–ligand pair. The protein–ligand complexes were prepared for these eleven ligands with two proteins, FIXa and FXa, as shown in Table 1. Therefore, a total of 22 protein–ligand complexes were used in this study.

### 3.2. Molecular Dynamics Simulation

In this study, all MD simulations were performed using GROMACS 2019.4 [47] with the all-atom amber99sb force field [48]. Ligand topologies and parameters were generated using Antechamber [49], and the charges were determined using the AM1-BCC [50] method. The system was neutralized by adding Na^+^ and Cl^−^ ions at a concentration of 0.15 mol/L. The bonds were constrained using the LINCS algorithm [51]. For long-range electrostatic interactions, the particle mesh Ewald (PME) method [52] was employed. A cutoff of 12 Åwas applied for both Van der Waals and short-range electrostatic interactions. The simulation time step was set to 2 fs.

MD simulations were performed in a step-by-step process, starting with minimization, followed by NVT (1 ns), NPT with restraints (1 ns), NPT without restraints (10 ns), and production (100 ns) runs. During minimization, NVT, and the first NPT equilibration, positional restraints were applied to the heavy atoms of both the protein and ligand utilizing a harmonic potential. Energy minimization was achieved using the steepest descent algorithm implemented in GROMACS. Throughout NVT and the first NPT equilibration, the temperature was controlled using the V-rescale method, set at 310 K. Additionally, a constant pressure of 1 bar was set using the Berendsen method [53] in first NPT equilibration run. Then, unrestrained NPT simulations were performed for 10 ns. Production simulations were run for 100 ns, and each was repeated three times. During the unrestrained NPT and production simulations, the temperature was controlled using the Nosé–Hoover thermostat [54,55], while the pressure was controlled utilizing the Parinello–Rahman barostat [56]. Basic analysis was carried out using VMD [57].

### 3.3. Computational Analysis

#### 3.3.1. The Movement Level of Ligands

The long inhibitor binding sites of FIXa and FXa were divided into four subsites (Figure 1a): S1, S2, S3, and S4. The ligands were also divided into four parts, P1, P2, P3, and P4, (Figure 1b), corresponding to the inhibitor binding site subsites to which they bind [3]. To examine the ligand dynamics, three atoms (marked in red, green, and yellow in Figure S7), located at P1, P2, and P4 were chosen, and their coordinates were extracted throughout the entire simulation from three independent replicate runs. The extracted coordinates were overlaid onto the initial protein–ligand complex, enabling evaluation of the protein–ligand binding stability by assessing the displacement of each atom during the simulation.

#### 3.3.2. Contact Frequency Calculation

Two types of protein–ligand contacts were calculated throughout the simulation using the *contactFreq.tcl* script of VMD. Hydrogen bond contacts were determined by analyzing identified hydrogen bond pairs within a cutoff distance of 3.5 Å using the *gmx hbond* tool in GROMACS. van der Waals contacts were calculated within a cutoff distance of 4 Å. For the analysis, only contacts that occurred in more than 40% of the entire trajectory were included.

#### 3.3.3. Binding Free-Energy Calculation

The binding free energy ($\Delta G_{bind}$) of the protein–ligand complex was calculated using the molecular mechanics Poisson–Boltzmann surface area (MMPBSA) method [58].

(1) $$\Delta G_{bind}=G_{complex}-\left(G_{protein}+G_{ligand}\right)$$

The free energy of each individual entity is given by

(2) $$\Delta G_{x}=<E_{MM}>+<G_{solv}>-TS$$

where *x* is the protein–ligand complex, protein, or ligand. $<E_{MM}>$ and $<G_{solv}>$ are the average molecular mechanics energy and the average solvation free energy, respectively. $<G_{solv}>$ is separated into polar and non-polar terms. It is calculated using an implicit solvent model. The polar terms are computed using the Poisson–Boltzmann (PB) equation, while the non-polar term is determined based on the solvent accessible surface area (SASA) model.

To calculate the binding free energy, the g_mmpbsa package, developed by the Open Source Drug Discovery Consortium [59], was utilized. This package enables binding free-energy determination, excluding the entropic term, as well as an evaluation of the energetic contributions of individual residues to the binding through an energy decomposition scheme. The binding free energy and contribution energy analyses were performed every 0.1 ns using the final 10 ns (in total 100 frames from each run) of the 100 ns production simulation. For the SASA approximation, the grid spacing was set to 0.5 Å and a probe radius of 1.4 Å was used. The solvent dielectric constant was set to 80, while the solute dielectric constant was set to 2.

#### 3.3.4. Binding Site Volume Calculation

The binding site volumes of FIXa and FXa complexed with the 11 ligands were calculated at 1 ns intervals during the MD simulations using Povme 3.0 [60]. A comparative analysis was performed to examine the dynamic variations of the binding sites when different ligands were bound to both FIXa and FXa.

#### 3.3.5. Protein–Ligand Interaction Fingerprint (IFP)

The interaction fingerprint (IFP) was calculated utilizing the Python package ProLIF v.1.0.0 [61]. This tool enables computing various types of interactions, such as HBAcceptor, HBDonor, hydrophobic, pi–pi stacking, cationic, and anionic interactions, from the MD trajectory. The IFP was calculated at 0.1 ns intervals in each run of the three replicated 100 ns simulations.

## 4. Conclusions

The conclusion of this current work is consistent with that of our previous work (Kundu et al. (2021) [3]); however, detailed dynamics profiles of the major protein–ligand interactions provide further insights into interaction stabilities. A number of minor interactions were also identified in this study which play an important role statistically in determining whether the overall binding is favorable.

The interaction fingerprint (IFP) analysis clearly reveals that formation of a strong hydrogen bond between the ligand and D189 and A190 at the S1 subsite is crucial for FIXa selectivity, consistent with the conclusion of our previous study (Kundu et al. (2021) [3]). This dynamic analysis identifies several more interactions that are specific to FIXa (see Tables S5 and S6). The absence of polar interactions contributes to the selectivity of FXa, which is another important finding obtained from the interactions’ dynamic profiles (see Table S6). Additional confirmation of the selectivity criteria for FIXa and FXa, as well as some criteria for binding and activity, is provided by the contact frequency analysis of the protein–ligand complexes (Figure 3 and Figure 4). The movement of the key interactions emphasizes that selective ligands are bound more tightly to the protein than non-selective and inactive ligands (Figure 2). MM-PBSA and binding site volume analyses were performed to determine binding affinities, and although limited, proteins with a high affinity for each ligand were distinguished. A residue-wise energy contribution analysis of MM-PBSA was provided to identify the specific residues located near the binding site, with the results consistent with other analyses. Molecular dynamics simulation provided a detailed overview of the binding interactions between the two coagulation factors, FIXa and FXa, and their respective inhibitors. Our findings shed light on the structural and dynamic basis for selective inhibition of FIXa over FXa and offer insights into potential strategies to develop new treatments for blood clotting disorders.

## Figures and Tables

**Table 1:** The experimental IC_50_ data and PDB ID of eleven ligands.

Dataset	Compound	hFIXa IC_50_ (nM)	hFXa IC_50_ (nM)	PDB ID (FIXa)	PDB ID (FXa)
Dataset 1 ^a^ FIXa selective	**1**	4.9	31,000	5TNT	-
	**2**	98	>100,000	5TNO	-
	**3**	172	>100,000	-	-
Dataset 2 FXa selective	**4** ^b^	Inactive	0.08	-	2P16
	**5** ^c^	Inactive	0.7	-	2W26
	**6** ^d^	Inactive	3.5	-	3KL6
Dataset 3 ^e^ Active for both FIXa and FXa	**7**	3.6	105	4ZAE	-
	**8**	8	195	-	-
	**9**	14.8	245	-	-
Dataset 4 Inactive for both FIXa and FXa	**10** ^a^	>3000	>100,000	-	-
	**11** ^f^	9000	62,500	4YZU	-

^a^ Sakurada et al. (2017) [19]. ^b^ Pinto et al. (2007) [13]. ^c^ Roehrig et al. (2005) [15]. ^d^ Fujimoto et al. (2010) [17]. ^e^ Zhang et al. (2015) [22]. ^f^ Parker et al. (2015) [20].

## Data Availability

Not applicable.

## Supplementary Materials

The following supporting information can be downloaded at: https://www.mdpi.com/article/10.3390/molecules28196909/s1, Figure S1: The cascading mechanism of the blood coagulation process; Figure S2: RMSD profiles of protein Cα (red) and heavy ligand atoms (black) during the entire trajectory of three replicated 100 ns MD simulations; Figure S3: The average RMSF plot with standard deviation of heavy chain during three replicated 100 ns simulations; Figure S4: Average protein–ligand binding free energy in the last 10 ns of three replicated 100 ns simulations; Figure S5: Average binding site volume of FIXa (yellow) and FXa (green) of three replicated simulations on each system; Figure S6: The crystal structures and ligand binding sites of factor IXa and factor Xa; Figure S7: Selected atoms for ligand dynamic analysis; Table S1: The top 10 residues regarding residual contribution energy between 11 ligands and FIXa; Table S2: The top 10 residues regarding residual contribution energy between 11 ligands and FXa; Table S3: The protein–ligand interactions of FIXa and FXa complexes during dynamic simulations; Table S4: The protein–ligand interactions of co-crystal structure from Kundu et. al. (2021); Table S5: The protein–ligand interactions of FIXa complexes with more than 40% occupancy during dynamic simulations; Table S6: The protein–ligand interactions of FXa complexes with more than 40% occupancy during the dynamic simulations.

## Sample Availability

Samples of the compounds are not available from the authors.

## Abbreviations

The following abbreviations are used in this manuscript:

DOAC	Direct Oral Anticoagulants
FIXa	Activated Coagulation Factor IX
FXa	Activated Coagulation Factor X
