# Supplementary material for: Molecular Dynamics Simulation Study of the Selective Inhibition of Coagulation Factor IXa over Factor Xa
Source: Molecules. 2023 Oct 2;28(19):6909. doi: 10.3390/molecules28196909 (PMC10574344; doi:10.3390/molecules28196909)
Supplement: Supplementary file 1 [file molecules-28-06909-s001.zip › molecules-2569807-supplementary.pdf]

Supplementary Materials: Molecular Dynamics Simulation Study of the Selective Inhibition of Coagulation Factor IXa over Factor Xa

Hyun Jung Yoon 1, Sibsankar Kundu 2 and Sangwook Wu 1,2,*

[1] Department of Physics, Pukyong National University, Busan 48513, Republic of Korea; hjyoon@pukyong.ac.kr
[2] R&D Center, PharmCADD Co., Ltd., Busan, 48792, Republic of Korea; sskundu@gmail.com
[*] Correspondence: sangwoow@pknu.ac.kr

Blood coagulation, essential for hemostasis, is a complex physiological process that ensures clot formation upon blood vessel injury, preventing excessive bleeding and maintaining normal blood flow under physiological conditions. Hemostasis involves a cascade of enzymatic reactions, with multiple target proteins belonging to the S1A sub-family of serine proteases, collectively referred to as coagulation factors. Figure S1 provides a concise overview of the basic coagulation mechanism. The complex coagulation process is operated through a cascading mechanism, subdivided into three smaller pathways, namely the intrinsic pathway, extrinsic pathway, and common pathway. The intrinsic pathway is triggered by blood vessel injury, while the extrinsic pathway is triggered by tissue injury. Downstream propagation of the intrinsic pathway involves the conversion of FXII to FXIIa, FXI to FXIa, and FIX to FIXa, while the extrinsic pathway propagates through the conversion of FVII to FVIIa with the assistance of tissue factor (TF). These two pathways merge into a common pathway, converting FX to FXa, and continue downstream with the conversion of to thrombin, ultimately leading to fibrillation or fibrin clot formation. FIXa and FXa are indicated by red enclosing boxes in Figure S1, illustrating how FIXa selectively regulates the intrinsic pathway without affecting the extrinsic pathway.

Direct and Indirect Regulation : The earliest therapies used as anticoagulants for thromboembolic disorders are heparin and warfarin, but these agents regulate the coagulation process through the indirect pathway and have limited use due to their bleeding risk [1–3] and in the event of emergencies. Therefore, the better therapeutic strategy has emerged in the form of Direct Oral AntiCoagulants (DOACs), which are also known as Novel Oral AntiCoagulants (NOACs) or Non-vitamin K mediated Oral AntiCoagulants (not indirect).

Descripton of long activesite and long ligand : The inhibitor binding sites or active sites in FIXa and FXa are considerably long. To facilitate the study and analysis of structure-activity relationships (SAR) and their pharmacological behavior, the long active site is divided into several subsites, specifically denoted as S1, S2, S3, and S4. Similarly, the extended ligand that binds to this active site and extends from the S1 subsite through S2 and S3 up to the S4 subsite, is also segmented into corresponding fragments denoted as P1, P2, P3, and P4.

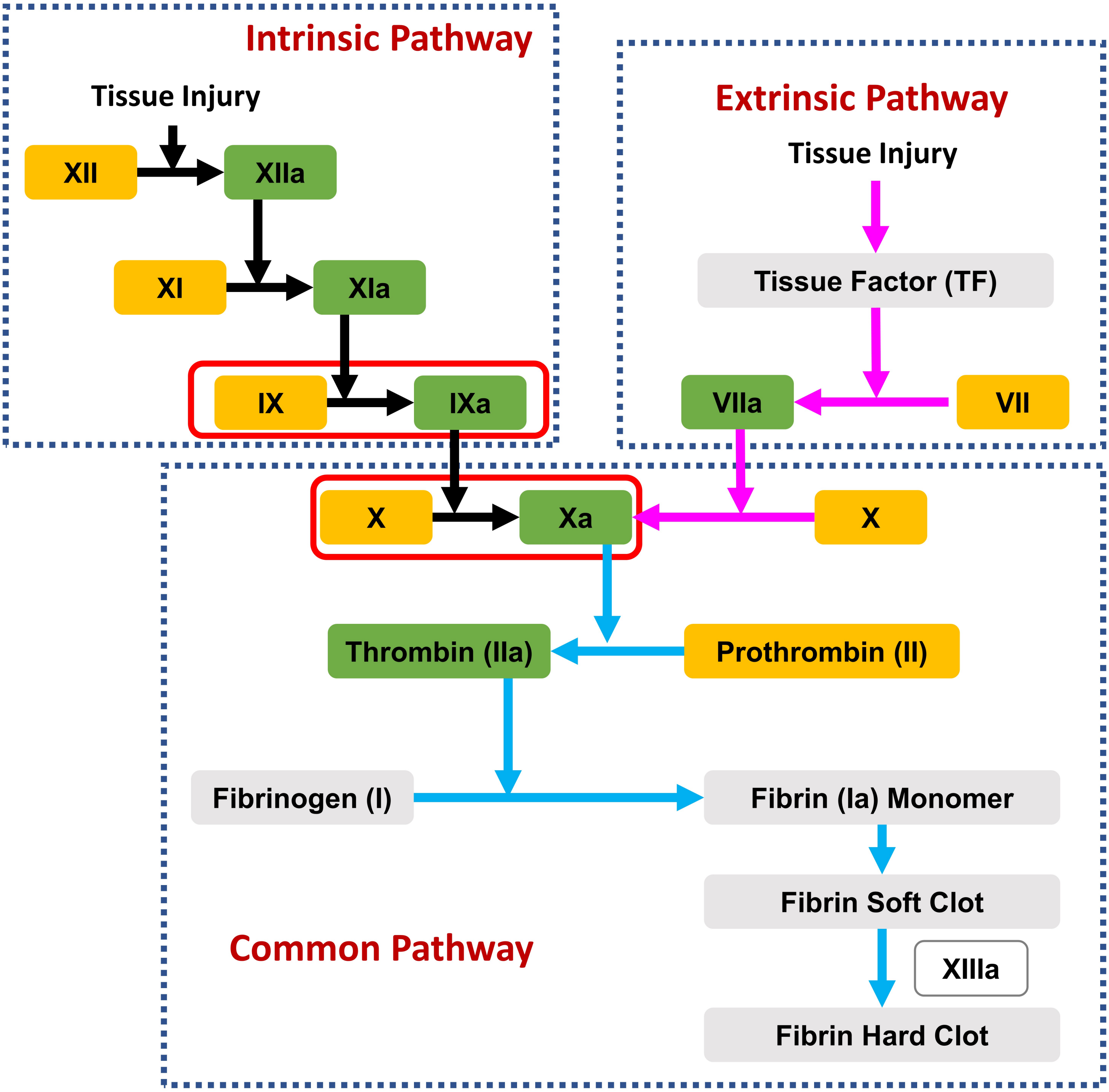

Figure S1. The cascading mechanism of blood coagulation process. The figure shows that FXa is in the first step of common pathway and FIXa in under the intrinsic pathway. The Activated and inactive factors are marked in green and orange, respectively.

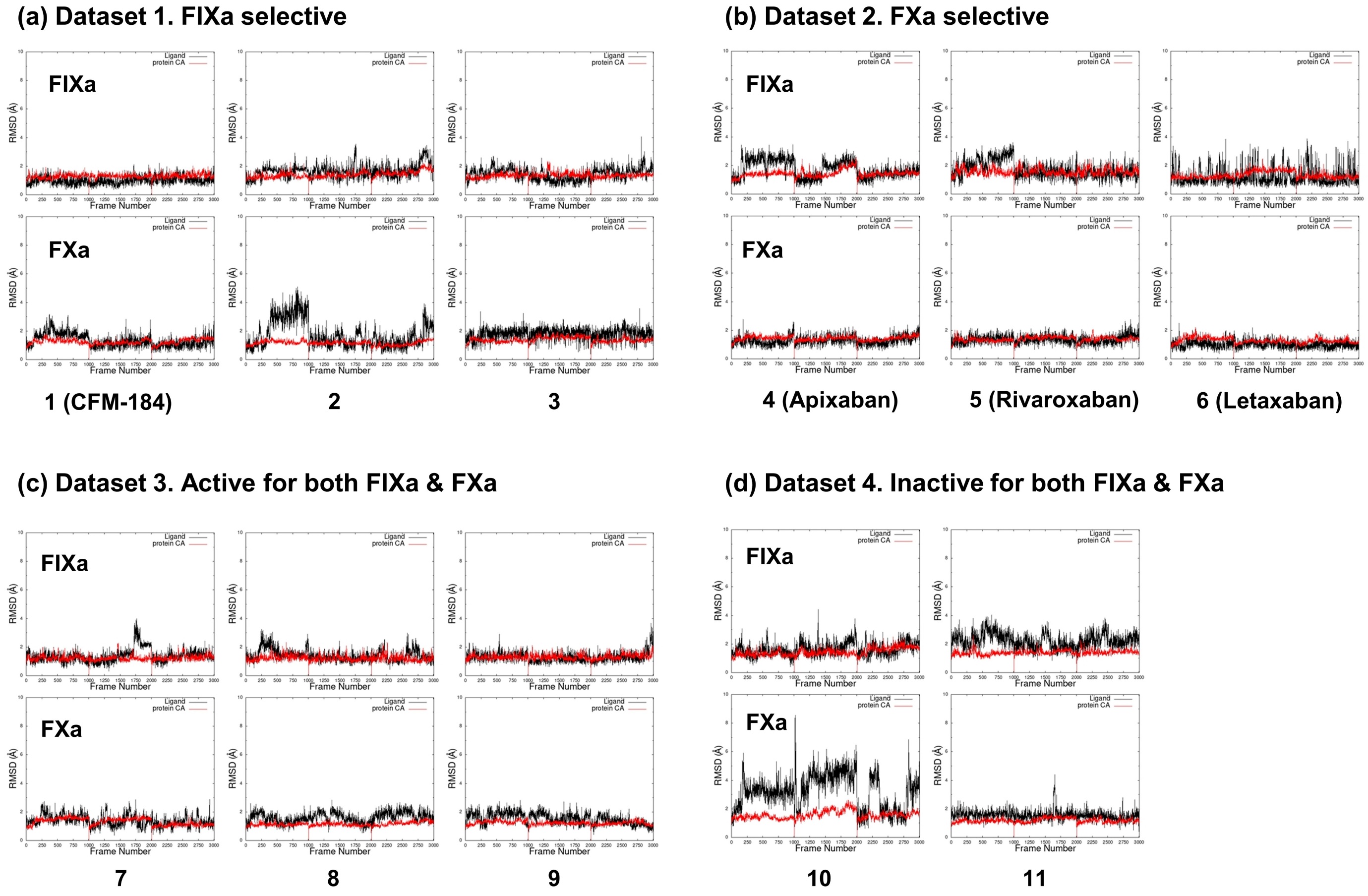

Figure S2. RMSD profiles of protein C_α (Red) and ligand heavy atom (Black) during entire trajectory of 3 replicated 100ns MD simulations (0-1000 : Run 1, 1001 - 2000 : Run 2, 2001-3000 : Run 3). All structure aligned to protein C_α atoms of first frame, and then calculated RMSD.

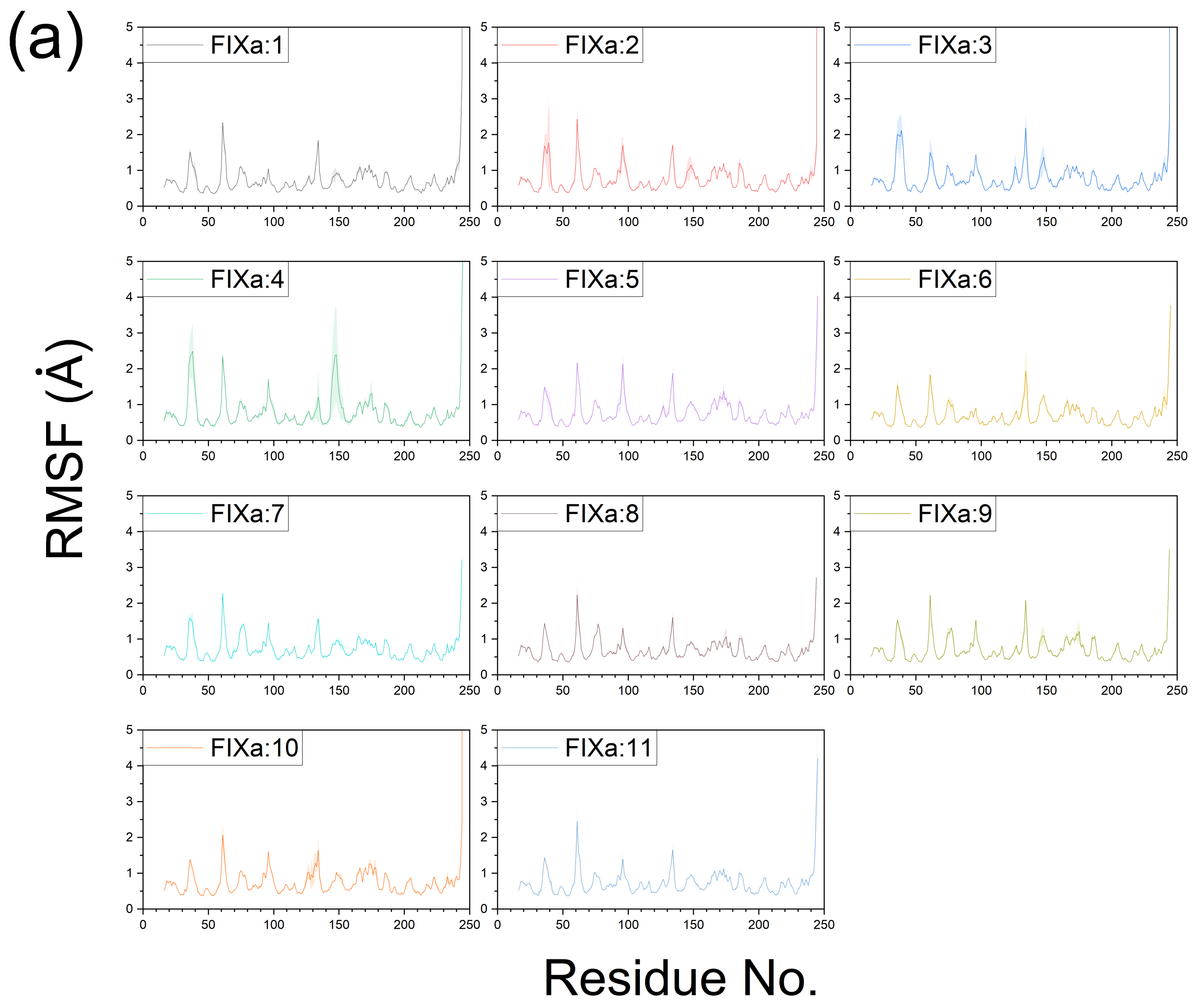

(a) The RMSF plots of the FIXa complexes.

Figure S3. The average RMSF plots with standard deviation of heavy chain during 3 replicated 100 ns simulations.

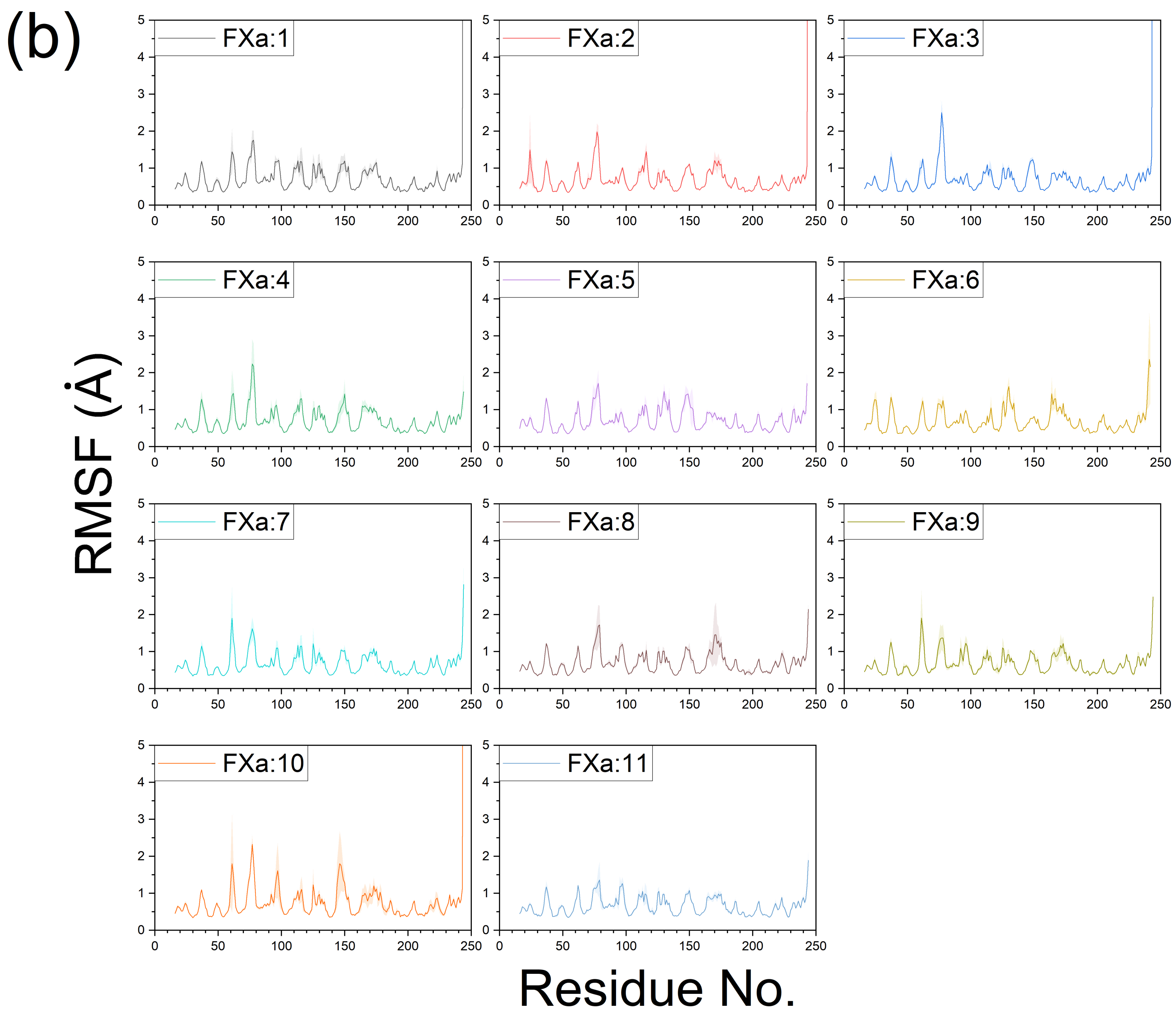

(b) The RMSF plots of the FXa complexes.

Figure S3. The average RMSF plots with standard deviation of heavy chain during 3 replicated 100 ns simulations.

Data 1 : FIXa selective								
FIXa:1			FIXa:2			FIXa:3		
Residue	Average (kJ/mol)	StDev (+/-)	Residue	Average (kJ/mol)	StDev (+/-)	Residue	Average (kJ/mol)	StDev (+/-)
G216	-10.826	0.333	W215	-15.643	2.231	G216	-11.843	0.607
W215	-10.665	0.455	F174	-10.906	0.248	F174	-11.005	0.585
Y99	-10.206	0.900	Y99	-10.277	0.505	W215	-9.780	0.703
F174	-9.693	0.504	Q192	-8.037	1.887	Y99	-7.666	1.439
C220	-8.401	0.432	G216	-6.887	2.798	C220	-6.937	0.281
Q192	-8.037	1.062	C220	-5.352	0.664	C191	-5.701	0.317
C191	-4.736	0.242	K224	-4.946	0.458	K224	-5.617	0.064
E217	-4.535	0.513	C191	-4.917	0.759	Q192	-4.207	0.423
K98	-4.154	0.323	K148	-4.141	1.892	E217	-4.155	1.384
K224	-4.058	0.015	K173	-3.505	1.891	D102	-2.847	1.025
**Data 2 : FXa selective**								
**FIXa:4**			**FIXa:5**			**FIXa:6**		
Residue	Average (kJ/mol)	StDev (+/-)	Residue	Average (kJ/mol)	StDev (+/-)	Residue	Average (kJ/mol)	StDev (+/-)
W215	-24.333	0.257	K173	-14.331	9.328	G216	-12.642	0.481
Y99	-7.839	0.189	W215	-10.669	1.503	F174	-9.684	0.172
Q192	-7.125	1.729	F174	-9.746	0.331	K148	-9.343	0.998
F174	-6.793	0.164	Y99	-9.028	1.132	K98	-8.975	0.829
C191	-6.200	1.168	Q192	-7.678	1.734	Y99	-7.845	1.221
C220	-3.957	0.528	K224	-5.883	1.939	K173	-6.248	1.511
G216	-3.811	0.460	T172	-3.422	3.861	R143	-4.558	0.723
K98	-3.342	0.583	C220	-2.883	0.460	W215	-4.020	0.347
K148	-2.679	1.231	I227	-2.408	3.101	Q192	-3.469	0.378
Y228	-2.252	1.058	K98	-1.840	2.386	C220	-3.380	0.298
**Data3 : Active for both FIXa & Fxa**								
**FIXa:7**			**FIXa:8**			**FIXa:9**		
Residue	Average (kJ/mol)	StDev (+/-)	Residue	Average (kJ/mol)	StDev (+/-)	Residue	Average (kJ/mol)	StDev (+/-)
W215	-15.718	0.152	W215	-15.124	0.097	W215	-15.515	0.399
Q192	-12.705	4.471	F174	-11.249	0.188	F174	-10.787	0.424
F174	-11.790	0.276	G216	-8.833	0.352	Q192	-9.250	1.808
G216	-10.152	0.843	Q192	-8.447	0.989	Y99	-9.094	0.996
Y99	-9.817	1.396	Y99	-8.052	1.051	G216	-8.691	0.632
C191	-5.967	2.806	C191	-7.036	0.603	C191	-5.783	2.097
K224	-3.592	0.428	C220	-3.788	0.252	C220	-3.353	0.670
C220	-3.461	0.611	V16	-2.628	0.560	K224	-2.837	0.181
K148	-2.653	0.464	K224	-2.071	0.223	V16	-2.814	0.418
V16	-2.370	0.456	E217	-1.994	0.487	I213	-2.169	0.088

Data4 : Inactive for both FIXa & Fxa					
FIXa:10			FIXa:11		
Residue	Average (kJ/mol)	StDev (+/-)	Residue	Average (kJ/mol)	StDev (+/-)
G216	-9.349	0.864	W215	-13.004	0.569
Q192	-7.205	0.455	F174	-10.513	0.380
F174	-6.666	1.385	Q192	-8.539	1.215
C220	-6.510	0.627	Y99	-8.494	1.424
K224	-6.257	0.905	C191	-5.529	0.611
K173	-5.636	0.899	K98	-5.020	0.084
K148	-4.606	0.414	G216	-4.538	0.603
C191	-4.413	0.604	C220	-4.095	0.540
Y99	-4.391	1.704	E217	-3.596	0.667
W215	-4.130	0.390	E219	-2.313	0.189

Table S1. The highest top 10 residues of residual contribution energy between 11 ligands and FIXa.

Data 1 : FIXa selective								
FXa:1			FXa:2			FXa:3		
Residue	Average (kJ/mol)	StDev (+/-)	Residue	Average (kJ/mol)	StDev (+/-)	Residue	Average (kJ/mol)	StDev (+/-)
W215	-11.498	0.899	W215	-10.979	4.967	E217	-11.297	0.736
G216	-11.244	0.673	G216	-9.498	1.854	G216	-10.967	0.253
C220	-8.008	0.126	F174	-7.921	2.994	W215	-7.930	0.267
Y99	-6.925	1.464	G218	-6.207	0.552	C220	-6.610	0.243
E217	-6.843	2.168	C220	-5.399	0.869	G218	-5.422	0.160
Q192	-5.628	0.369	C191	-5.298	0.483	Q192	-4.945	0.191
F174	-4.911	1.554	Q192	-4.639	1.293	C191	-4.499	0.175
C191	-4.404	0.149	E217	-4.358	2.464	G226	-3.670	0.150
G218	-3.852	0.317	Y99	-4.139	3.095	A190	-3.012	0.716
A190	-3.775	0.336	K224	-3.446	0.076	F174	-2.558	0.069
**Data 2 : FXa selective**								
**FXa:4**			**FXa:5**			**FXa:6**		
Residue	Average (kJ/mol)	StDev (+/-)	Residue	Average (kJ/mol)	StDev (+/-)	Residue	Average (kJ/mol)	StDev (+/-)
W215	-24.941	0.096	W215	-10.367	1.067	G216	-14.580	0.115
Y99	-7.784	0.541	F174	-9.237	0.359	W215	-9.641	0.875
Q192	-5.785	0.184	Y99	-8.241	0.355	E217	-7.703	0.528
C220	-5.473	1.188	G219	-6.472	2.093	Q192	-7.544	0.530
F174	-5.393	2.739	C220	-5.306	0.252	F174	-7.448	0.267
C191	-4.840	1.556	Q192	-4.765	1.288	R143	-6.133	0.176
K147	-4.556	0.211	R222	-4.295	1.410	K147	-5.929	0.276
E217	-3.728	0.093	E217	-4.256	1.403	Y99	-5.750	0.374
V213	-2.961	0.284	K96	-2.363	0.295	G219	-4.931	0.429
K96	-2.663	0.718	T98	-2.296	0.515	I16	-4.362	0.145
**Data3 : Active for both FIXa & Fxa**								
**FXa:7**			**Fxa:8**			**FXa:9**		
Residue	Average (kJ/mol)	StDev (+/-)	Residue	Average (kJ/mol)	StDev (+/-)	Residue	Average (kJ/mol)	StDev (+/-)
W215	-15.345	0.397	W215	-16.429	0.317	W215	-14.571	0.896
F174	-10.576	0.894	F174	-10.369	0.269	F174	-10.420	0.862
G216	-10.319	0.518	G216	-9.234	0.399	G216	-9.652	0.388
C191	-6.401	0.259	Q192	-8.174	0.762	E217	-6.977	0.702
Q192	-6.384	0.174	Y99	-7.442	1.968	Y99	-6.616	0.785
Y99	-5.922	0.466	E217	-7.400	0.688	Q192	-5.215	2.139
E217	-4.952	0.290	C191	-5.139	1.481	C191	-4.380	1.763
C220	-4.715	0.218	C220	-3.783	0.451	G218	-4.353	0.822
R222	-3.098	0.319	G218	-2.738	0.638	C220	-4.218	0.419
G218	-2.804	0.454	V213	-2.456	0.108	V213	-1.863	0.510

Data4 : Inactive for both FIXa & Fxa					
FXa:10			FXa:11		
Residue	Average (kJ/mol)	StDev (+/-)	Residue	Average (kJ/mol)	StDev (+/-)
G216	-11.067	0.541	W215	-12.510	0.361
W215	-7.723	1.796	F174	-10.059	0.458
C220	-6.588	1.636	Q192	-8.608	0.343
Q192	-6.224	0.218	E217	-6.204	0.268
E217	-5.116	2.336	E146	-6.131	0.170
C191	-4.467	0.504	Y99	-5.589	1.656
G218	-3.807	1.365	G216	-5.294	0.346
A190	-3.794	1.547	C220	-5.011	0.252
G226	-3.543	0.284	C191	-4.845	0.169
S173	-3.096	1.430	G218	-2.695	1.398

Table S2. The highest top 10 residues of residual contribution energy between 11 ligands and FXa.

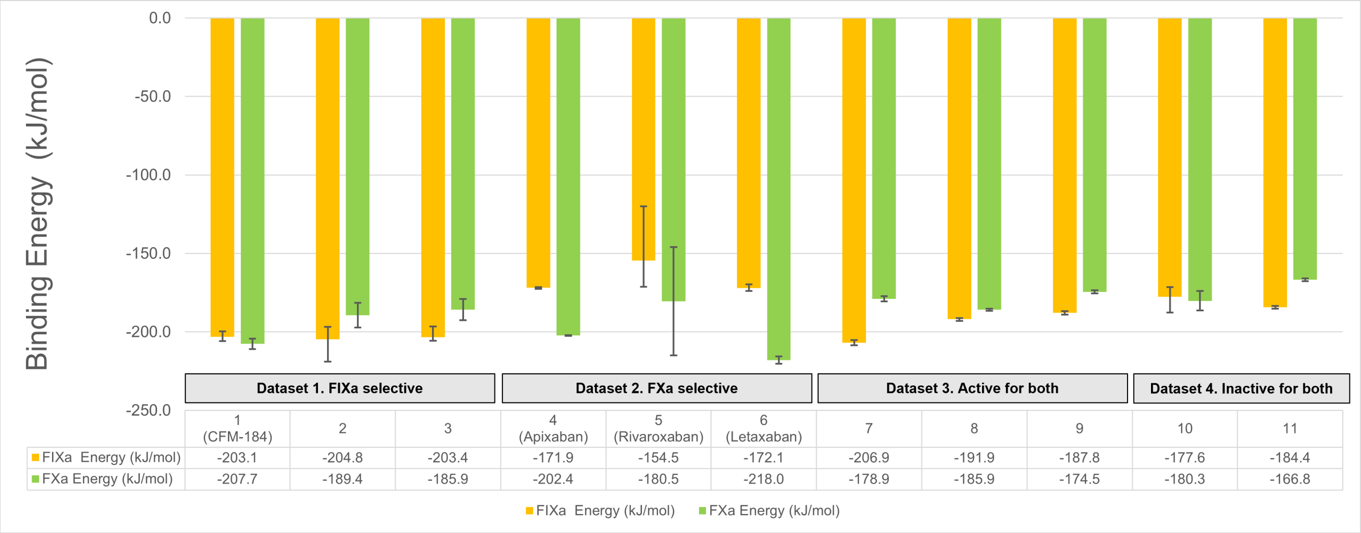

	1 (CFM-184)	2	3	4 (Apixaban)	5 (Rivaroxaban)	6 (Letaxaban)	7	8	9	10	11
FIXa Energy (kJ/mol)	-203.1	-204.8	-203.4	-171.9	-154.5	-172.1	-206.9	-191.9	-187.8	-177.6	-184.4
FXa Energy (kJ/mol)	-207.7	-189.4	-185.9	-202.4	-180.5	-218.0	-178.9	-185.9	-174.5	-180.3	-166.8

Figure S4. Average protein-ligand binding free energy on last 10ns simulation of 3 replicated 100ns simulations. The binding free energy between of ligand and FIXa marked in yellow, FXa marked in green.

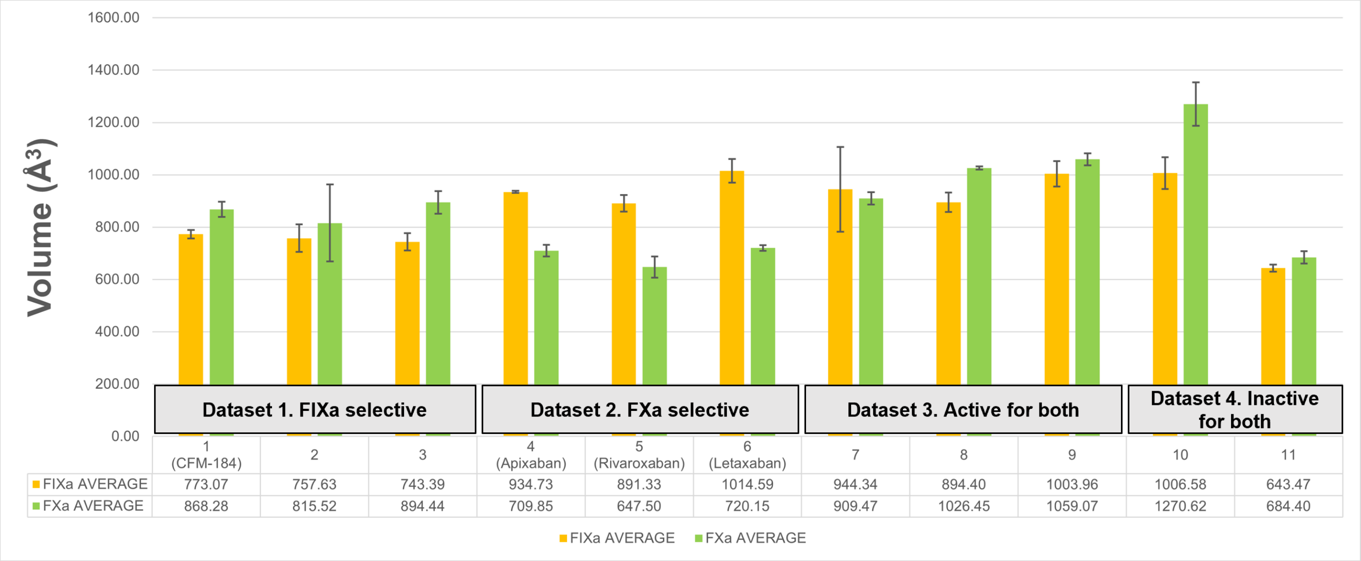

	1 (CFM-184)	2	3	4 (Apixaban)	5 (Rivaroxaban)	6 (Letaxaban)	7	8	9	10	11
FIXa AVERAGE	773.07	757.63	743.39	934.73	891.33	1014.59	944.34	894.40	1003.96	1006.58	643.47
FXa AVERAGE	868.28	815.52	894.44	709.85	647.50	720.15	909.47	1026.45	1059.07	1270.62	684.40

Figure S5. Average binding site volume of FIXa (Yellow) and FXa (Green) of 3 replicated simulations on each system.

Table S3. The protein-ligand interaction of FIXa and FXa complex during the dynamics.

Protein	Dataset	Compound	PDB ID	H-Bond						Pi-Pi Stacking			
FIXa	Dataset 1	1	5TNT	D189<br>0.78 (±0.08)	S190<br>0.68 (±0.05)	G216<br>0.79 (±0.02)				Y99<br>0.97 (±0.01)	F174<br>0.92 (±0.01)	W215<br>0.97 (±0.00)	
		2	5TNO	D189<br>0.68 (±0.19)	S190<br>0.80 (±0.07)	G216<br>0.69 (±0.17)				Y99<br>0.96 (±0.02)	F174<br>0.94 (±0.03)	W215<br>0.94 (±0.00)	
	Dataset 3	7	4ZAE			G216<br>0.97 (±0.05)				Y99<br>0.98 (±0.01)	F174<br>0.94 (±0.00)	W215<br>0.93 (±0.00)	H147<br>0.64 (±0.13)
	Dataset 4	11	4YZU			G216<br>0.84 (±0.03)				Y99<br>0.98 (±0.01)	F174<br>0.98 (±0.01)	W215<br>0.90 (±0.02)	
FXa	Dataset 2	4	2P16			G216<br>0.98 (±0.00)		E146<br>0.94 (±0.02)		Y99<br>0.66 (±0.02)		W215<br>0.53 (±0.07)	
		5	2W26				G218/219<br>0.95 (±0.02)			Y99<br>0.56 (±0.05)	F174<br>0.64 (±0.03)	W215<br>0.55 (±0.03)	
		6	3KL6			G216<br>1.00 (±0.00)	G218/219<br>0.77 (±0.03)		E97<br>0.74 (±0.02)				

Table S4. The protein-ligand interaction of co-crystal structure from Kundu et. al. (2021) [3].

Protein	Dataset	Compound	PDB ID	H-Bond							Halogen Bond	Pi-Pi Stacking		
FIXa	Dataset 1	1	5TNT	D189	S190	G216	-	-	-	-	G216	F174	-	-
		2	5TNO	D189	S190	G216	-	-	-	-	G216	-	-	-
	Dataset 3	7	4ZAE	-	-	G216	-	-	-	-	G216	F174	-	-
	Dataset 4	11	4YZU	-	-	-	-	-	-	-	-	F174	W215	-
FXa	Dataset 2	4	2P16	-	-	G216	-	E146	-	-	-	-	-	Y99
		5	2W26	-	-	-	G219	-	-	-	-	-	-	Y99
		6	3KL6	-	-	G216	G219	-	Q192	E97	-	-	-	Y99

Table S5. The protein-ligand interactions of FIXa complex more than 40% occupancy during the dynamics. The highlighted color blocks represent interaction type (Green: Common interaction with all ligands, Yellow: common interaction with active ligads, Oragne: Interaction with only selective ligands, Blue: Interaction with active ligands without selectivity).

Subsite	Residue	Interaction	Dataset 1. FIXa selective			Dataset 2. FXa selective			Dataset 3. Active for botn FIXa & FXa			Dataset 4. Inactive for botn FIXa & FXa	
			FIXa:1	FIXa:2	FIXa:3	FIXa:4	FIXa:5	FIXa:6	FIXa:7	FIXa:8	FIXa:9	FIXa:10	FIXa:11
			Average	Average	Average	Average	Average	Average	Average	Average	Average	Average	Average
S4	I96	HBDonor	-	-	-	-	-	0.68 (0.02)	-	-	-	-	-
S4	N97	Hydrophobic	0.64 (0.02)	-	-	-	-	0.97 (0.00)	-	-	-	-	-
S4	Y99	Hydrophobic	1.00 (0.00)	1.00 (0.00)	1.00 (0.00)	1.00 (0.00)	1.00 (0.00)	1.00 (0.00)	1.00 (0.00)	1.00 (0.00)	1.00 (0.00)	0.97 (0.01)	1.00 (0.00)
S4	Y99	PiStacking	0.97 (0.01)	0.96 (0.02)	0.97 (0.01)	-	0.61 (0.07)	-	0.98 (0.01)	0.95 (0.02)	0.95 (0.03)	0.80 (0.13)	0.98 (0.01)
S2	R143	Hydrophobic	-	-	-	-	-	-	0.81 (0.17)	0.87 (0.08)	0.93 (0.03)	-	-
S2	H147	Hydrophobic	0.89 (0.04)	-	-	-	-	-	0.84 (0.12)	0.83 (0.09)	0.85 (0.09)	0.57 (0.08)	0.41 (0.01)
S2	H147	PiStacking	-	-	-	-	-	-	0.64 (0.13)	0.65 (0.12)	0.70 (0.08)	-	-
S2	K148	Hydrophobic	-	-	-	-	-	-	0.80 (0.10)	0.87 (0.03)	0.84 (0.05)	-	-
S3-S4	F174	Hydrophobic	1.00 (0.00)	1.00 (0.00)	1.00 (0.00)	0.98 (0.02)	1.00 (0.01)	1.00 (0.00)	1.00 (0.00)	0.98 (0.03)	0.92 (0.07)	0.99 (0.01)	1.00 (0.00)
S3-S4	F174	PiStacking	0.92 (0.01)	0.94 (0.03)	0.97 (0.00)	0.50 (0.08)	0.67 (0.13)	-	0.94 (0.00)	0.87 (0.06)	0.86 (0.06)	0.89 (0.10)	0.98 (0.01)
	Y177	Hydrophobic	-	-	-	0.51 (0.03)	-	-	-	-	-	-	-
S1	D189	HBDonor	0.78 (0.08)	0.68 (0.19)	0.86 (0.04)	-	-	-	-	-	-	0.85 (0.05)	-
S1	D189	Hydrophobic	-	-	0.62 (0.07)	-	-	-	-	-	-	-	-
S1	S190	Hydrophobic	1.00 (0.00)	0.98 (0.01)	1.00 (0.00)	0.98 (0.01)	0.97 (0.01)	0.84 (0.03)	0.99 (0.01)	1.00 (0.00)	1.00 (0.00)	1.00 (0.00)	1.00 (0.00)
S1	S190	HBDonor	0.68 (0.05)	0.80 (0.07)	-	-	-	-	-	-	-	0.59 (0.06)	-
S1	C191	Hydrophobic	1.00 (0.00)	1.00 (0.00)	1.00 (0.00)	1.00 (0.00)	1.00 (0.00)	1.00 (0.00)	1.00 (0.00)	1.00 (0.00)	1.00 (0.00)	1.00 (0.00)	1.00 (0.00)
S1	Q192	Hydrophobic	0.99 (0.00)	1.00 (0.00)	1.00 (0.00)	1.00 (0.00)	1.00 (0.00)	0.99 (0.00)	1.00 (0.00)	1.00 (0.00)	1.00 (0.00)	0.99 (0.01)	1.00 (0.00)
S1	S195	Hydrophobic	-	-	-	-	-	-	0.97 (0.01)	0.97 (0.01)	0.99 (0.00)	-	0.93 (0.05)
S1	I213	Hydrophobic	0.93 (0.02)	-	0.86 (0.07)	0.99 (0.00)	0.81 (0.06)	0.97 (0.00)	0.99 (0.01)	1.00 (0.00)	1.00 (0.00)	0.92 (0.03)	0.98 (0.01)
S1	S214	Hydrophobic	0.69 (0.02)	-	-	0.91 (0.02)	0.66 (0.12)	0.92 (0.02)	1.00 (0.00)	0.99 (0.00)	1.00 (0.00)	0.67 (0.03)	0.94 (0.04)
S1	W215	Hydrophobic	1.00 (0.00)	1.00 (0.00)	1.00 (0.00)	1.00 (0.00)	1.00 (0.00)	1.00 (0.00)	1.00 (0.00)	1.00 (0.00)	1.00 (0.00)	1.00 (0.00)	1.00 (0.00)
S1	W215	PiStacking	0.97 (0.00)	0.94 (0.00)	0.88 (0.03)	0.74 (0.09)	0.57 (0.10)	-	0.93 (0.00)	0.91 (0.01)	0.91 (0.01)	-	0.90 (0.02)
S1	G216	Hydrophobic	1.00 (0.00)	1.00 (0.00)	1.00 (0.00)	1.00 (0.00)	1.00 (0.00)	1.00 (0.00)	1.00 (0.00)	1.00 (0.00)	1.00 (0.00)	1.00 (0.00)	1.00 (0.00)
S1	G216	HBAcceptor	-	-	-	0.95 (0.02)	-	-	-	-	-	-	-
S1	G216	HBDonor	0.79 (0.02)	0.69 (0.17)	0.86 (0.03)	-	-	0.85 (0.01)	0.97 (0.05)	1.00 (0.00)	1.00 (0.00)	0.87 (0.02)	0.84 (0.03)
S1	E217	Hydrophobic	1.00 (0.00)	0.84 (0.13)	0.96 (0.01)	-	0.76 (0.05)	0.91 (0.01)	0.93 (0.04)	0.93 (0.02)	0.95 (0.03)	0.98 (0.01)	0.91 (0.04)
S1	E219	Hydrophobic	0.56 (0.04)	-	-	-	-	-	-	-	-	-	-
S1	C220	Hydrophobic	0.99 (0.00)	1.00 (0.00)	1.00 (0.00)	0.99 (0.01)	0.79 (0.09)	0.97 (0.00)	0.90 (0.11)	0.94 (0.05)	0.97 (0.03)	1.00 (0.00)	0.92 (0.03)
S1	G226	Hydrophobic	-	-	0.64 (0.05)	-	-	-	-	-	-	-	-

* represent that the compound has the co-crystal structures.

Table S6. The protein-ligand interactions of FXa complex more than 40% occupancy during the dynamics. The highlighted color blocks represent interaction type (Green: Common interaction with all ligands, Yellow: common interaction with active ligads, Oragne: Interaction with only selective ligands, Blue: Interaction with active ligands without selectivity).

Subsite	Residue	Interaction	Dataset 1. FIXa selective			Dataset 2. FXa selective			Dataset 3. Active for botn FIXa & FXa			Dataset 4. Inactive for botn FIXa & FXa	
			FXa:1	FXa:2	FXa:3	FXa:4	FXa:5	FXa:6	FXa:7	FXa:8	FXa:9	FXa:10	FXa:11
			Average	Average	Average	Average	Average	Average	Average	Average	Average	Average	Average
S4	E97	Hydrophobic	-	-	-	0.75 (0.11)	0.89 (0.01)	0.54 (0.04)	-	-	-	-	-
S4	E97	HBDonor	-	-	-	-	-	0.74 (0.02)	-	-	-	-	-
S4	T98	Hydrophobic	0.64 (0.13)	-	-	0.98 (0.01)	0.99 (0.00)	0.97 (0.01)	0.55 (0.08)	-	-	-	-
S4	Y99	Hydrophobic	0.98 (0.02)	0.95 (0.03)	0.93 (0.02)	1.00 (0.00)	1.00 (0.00)	1.00 (0.00)	1.00 (0.00)	1.00 (0.00)	1.00 (0.00)	-	1.00 (0.00)
S4	Y99	PiStacking	0.74 (0.05)	-	-	0.66 (0.02)	0.56 (0.05)	-	0.81 (0.02)	0.85 (0.04)	0.86 (0.04)	-	0.79 (0.07)
S2	R143	Hydrophobic	-	-	-	-	-	-	0.74 (0.11)	-	0.58 (0.12)	-	-
S2	E146	Hydrophobic	0.76 (0.01)	-	-	0.75 (0.03)	-	-	0.92 (0.01)	0.86 (0.05)	0.91 (0.02)	-	-
S2	E146	HBDonor	-	-	-	0.94 (0.02)	-	-	-	-	-	-	-
S3-S4	S173	Hydrophobic	-	-	0.79 (0.01)	-	-	-	-	-	-	-	-
S3-S4	F174	Hydrophobic	0.98 (0.01)	0.98 (0.02)	-	0.98 (0.01)	1.00 (0.00)	1.00 (0.00)	1.00 (0.00)	0.99 (0.00)	0.98 (0.02)	0.86 (0.11)	1.00 (0.00)
S3-S4	F174	PiStacking	0.77 (0.05)	0.87 (0.11)	-	-	0.64 (0.03)	-	0.91 (0.01)	0.86 (0.01)	0.86 (0.03)	-	0.96 (0.00)
S1	D189	HBDonor	0.91 (0.02)	0.64 (0.04)	0.91 (0.01)	-	-	-	-	-	-	0.80 (0.08)	-
S1	A190	Hydrophobic	1.00 (0.00)	0.97 (0.01)	1.00 (0.00)	0.89 (0.01)	0.97 (0.03)	1.00 (0.00)	0.99 (0.00)	0.85 (0.03)	0.97 (0.01)	0.99 (0.01)	0.99 (0.00)
S1	A190	HBDonor	0.55 (0.03)	0.71 (0.03)	0.50 (0.06)	-	-	-	-	-	-	0.57 (0.11)	-
S1	C191	Hydrophobic	1.00 (0.00)	1.00 (0.00)	1.00 (0.00)	1.00 (0.00)	0.99 (0.01)	1.00 (0.00)	1.00 (0.00)	0.97 (0.02)	0.99 (0.01)	0.99 (0.00)	1.00 (0.00)
S1	Q192	Hydrophobic	0.98 (0.01)	1.00 (0.00)	1.00 (0.00)	1.00 (0.00)	0.86 (0.11)	1.00 (0.00)	1.00 (0.00)	1.00 (0.00)	1.00 (0.00)	0.98 (0.00)	1.00 (0.00)
S1	S195	Hydrophobic	-	-	-	-	-	-	0.74 (0.07)	0.77 (0.04)	0.74 (0.09)	-	0.98 (0.01)
S1	V213	Hydrophobic	0.99 (0.00)	0.62 (0.05)	0.95 (0.04)	1.00 (0.00)	1.00 (0.00)	1.00 (0.00)	1.00 (0.00)	0.99 (0.01)	1.00 (0.00)	0.99 (0.00)	1.00 (0.00)
S1	S214	Hydrophobic	0.58 (0.13)	0.78 (0.03)	0.77 (0.08)	0.90 (0.03)	0.57 (0.09)	0.99 (0.00)	0.94 (0.01)	0.97 (0.01)	0.94 (0.03)	0.83 (0.03)	0.99 (0.00)
S1	W215	Hydrophobic	1.00 (0.00)	1.00 (0.00)	1.00 (0.00)	1.00 (0.00)	1.00 (0.00)	1.00 (0.00)	1.00 (0.00)	1.00 (0.00)	1.00 (0.00)	1.00 (0.00)	1.00 (0.00)
S1	W215	PiStacking	0.97 (0.01)	0.78 (0.18)	0.60 (0.04)	0.53 (0.07)	0.55 (0.03)	-	0.96 (0.02)	0.95 (0.01)	0.91 (0.05)	0.57 (0.13)	0.92 (0.01)
S1	G216	Hydrophobic	1.00 (0.00)	1.00 (0.00)	1.00 (0.00)	1.00 (0.00)	1.00 (0.00)	1.00 (0.00)	1.00 (0.00)	1.00 (0.00)	1.00 (0.00)	1.00 (0.00)	1.00 (0.00)
S1	G216	HBDonor	0.81 (0.02)	0.75 (0.08)	0.96 (0.01)	-	-	1.00 (0.00)	1.00 (0.00)	1.00 (0.00)	1.00 (0.00)	0.88 (0.04)	0.56 (0.10)
S1	G216	HBAcceptor	-	-	-	0.98 (0.00)	-	-	-	-	-	-	-
S1	E217	Hydrophobic	0.91 (0.02)	0.73 (0.09)	0.99 (0.00)	-	0.96 (0.02)	0.53 (0.06)	0.92 (0.03)	0.93 (0.03)	0.97 (0.02)	0.90 (0.08)	0.85 (0.07)
S1	G218/219	Hydrophobic	0.97 (0.01)	0.91 (0.01)	0.84 (0.01)	0.54 (0.12)	0.98 (0.01)	-	0.71 (0.05)	0.71 (0.07)	0.84 (0.04)	0.90 (0.02)	0.80 (0.03)
S1	G218/219	HBDonor	-	-	-	-	0.85 (0.09)	-	-	-	-	-	0.87 (0.04)
S1	G218/219	HBAcceptor	-	-	-	-	0.95 (0.02)	0.77 (0.03)	-	-	-	-	-
S1	C220	Hydrophobic	1.00 (0.00)	1.00 (0.00)	1.00 (0.00)	0.94 (0.08)	0.98 (0.02)	1.00 (0.00)	0.97 (0.01)	0.84 (0.10)	-	0.94 (0.09)	0.95 (0.01)
S1	G226	Hydrophobic	-	-	0.73 (0.06)	0.96 (0.01)	0.89 (0.13)	0.92 (0.01)	0.65 (0.06)	-	0.50 (0.07)	0.87 (0.11)	-
S1	I227	Hydrophobic	-	-	-	0.69 (0.08)	-	0.84 (0.03)	-	-	-	-	-
S1	Y228	Hydrophobic	-	-	-	0.87 (0.05)	0.72 (0.13)	0.96 (0.00)	-	-	-	-	-

* represent that the compound has the co-crystal structures.

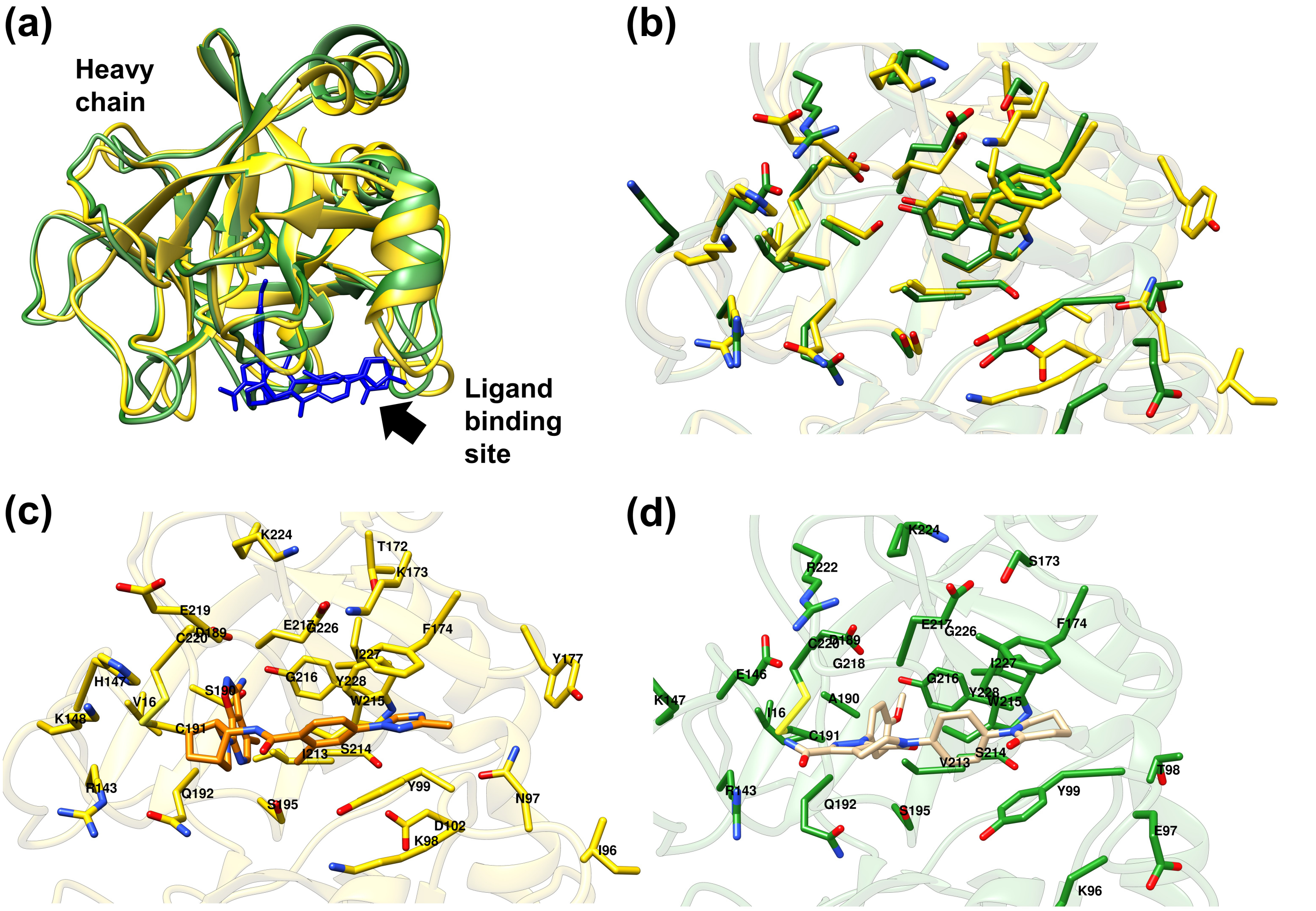

Figure S6. The crystal structure and ligand binding site of Factor IXa and Factor Xa. (a) The crystal structure of FIXa (Yellow, PDB 5TNT) and FXa (Green, PDB 2P16). (b) Superimposed binding site residues of FIXa (Yellow) and FXa (Green). (c) The binding site residues of FIXa and CFM-184 (Orange). (d) The binding site residues of FXa and Apixaban(Tan).

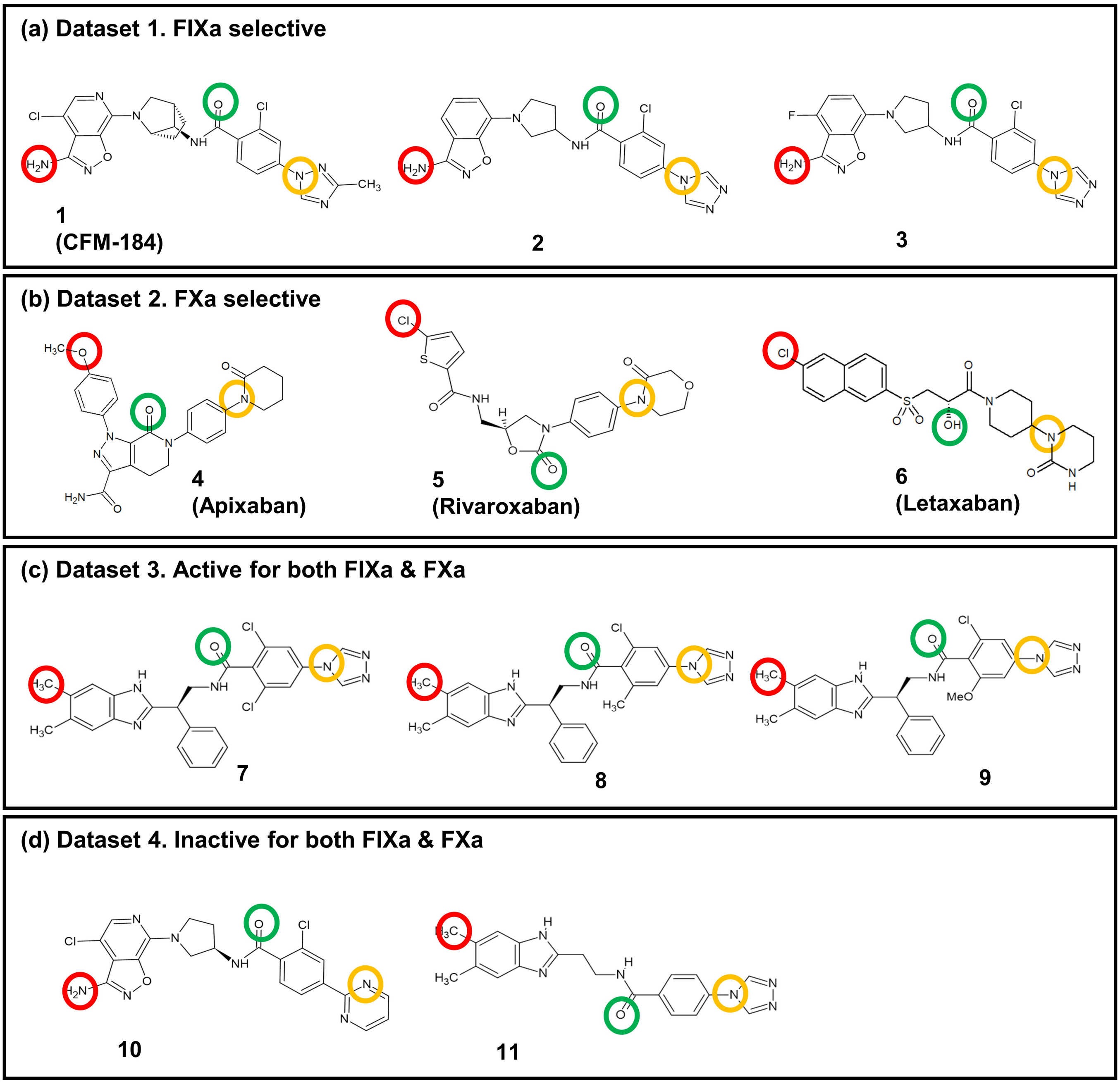

Figure S7. The selected atoms for ligand dynamic analysis. The red, green and yellow circle indicated ligand atoms of P1, P2 and P4, respectively.

References

1. Fischer, P.M. Design of Small-Molecule Active-Site Inhibitors of the S1A Family Proteases as Procoagulant and Anticoagulant Drugs. *Journal of Medicinal Chemistry* **2018**, *61*, 3799–3822. https://doi.org/10.1021/acs.jmedchem.7b00772.
2. Kabankin, A.S.; Sinauridze, E.I.; Lipets, E.N.; Ataullakhanov, F.I. Computer Design of Low-Molecular-Weight Inhibitors of Coagulation Factors. *Biochemistry (Moscow)* **2019**, *84*, 119–136. https://doi.org/10.1134/S0006297919020032.
3. Kundu, S.; Wu, S. A Structure Based Study of Selective Inhibition of Factor IXa over Factor Xa. *Molecules* **2021**, *26*, 5372. https://doi.org/10.3390/molecules26175372.
